# Risk factors accounting for anal incontinence during the first year after vaginal delivery—A case control study in China

**DOI:** 10.3389/fmed.2023.1073073

**Published:** 2023-05-02

**Authors:** Yang Jia, Qingao Liu, Lin Zeng, Yan Wang

**Affiliations:** ^1^Department of Obstetrics and Gynecology, Peking University Third Hospital, Beijing, China; ^2^Cuigezhuang Community Health Service Center, Beijing, China; ^3^Research Center of Clinical Epidemiology, Peking University Third Hospital, Beijing, China; ^4^National Center for Healthcare Quality Management in Obstetrics, Beijing, China; ^5^National Clinical Research Center for Obstetrics and Gynecology, Peking University Third Hospital, Beijing, China

**Keywords:** anal incontinence, vaginal delivery, postpartum, risk factors, prediction

## Abstract

**Introduction and hypothesis:**

Anal incontinence (AI) is a prevalent postpartum disorder. This study aims to investigate and quantify the risk factors for AI in the Chinese population during the first year following vaginal delivery.

**Methods:**

The case control study was conducted at Peking University Third Hospital, including all women who delivered vaginally between January 1, 2014, and June 30, 2018. Participants were followed up by telephone interviews 1 year after delivery. AI was defined as the involuntary loss of flatus or feces using a retrospective Jorge and Wexner score above 0. Clinical data were retrieved from the medical record system. Univariate and multivariate analyses were applied to identify potential risk factors accounting for AI. Based on the logistic regression model, a nomogram was constructed to predict the probability of AI postpartum. Restricted cubic spline was utilized to explore potential non-linear relationships between birth weight and AI postpartum.

**Results:**

Among the 140 AI and 421 none AI cases, we observed antepartum factors like every 100 g of birth weight gain (*OR* 1.39, *95% CI* 1.30–1.49), while intrapartum factors like forceps-assisted vaginal delivery (*OR* 7.11, *95% CI* 2.60–19.45), midline episiotomy (*OR* 13.11, *95% CI* 1.71–100.89), second-degree perineal tear (*OR* 6.51, *95% CI* 1.16–36.68), and third to fourth-degree perineal tear were independent risk factors for postpartum AI. Significantly, infant weighing over 3,400 g at birth increased the risk of AI postpartum. Based on logistic regression model, we constructed a nomogram to estimate the risk of AI 1 year after vaginal delivery.

**Conclusion:**

Our findings indicated that during the first year following vaginal delivery, infant with birth weight of 3,400 g or more, forceps-assisted vaginal delivery, midline episiotomy, and second to fourth-degree perineal tear increased the risk of AI. As a result, it is essential to limit the routine use of forceps and midline episiotomy and to monitor fetal weight during prenatal care.

## 1. Introduction

Anal incontinence (AI) is defined as the voluntary control loss of flatus or stool (1). The prevalence of AI ranges from 2 to 28% among women (2–4). In women who have suffered severe perineal tears, the prevalence of AI can be even higher, ranging from 17–62% (5). However, due to patients’ concerns about privacy and other issues, the actual prevalence rate of AI is believed to be higher than what’s presently reported. Therefore, AI is a challenging condition with negative psychosocial impacts and diverse etiologies that requires further investigation.

Anal incontinence is associated with increasing age, newborn weight, parity, instrumental vaginal delivery, and anal sphincter injury (6). Obstetric factors, particularly intrapartum factors, also contribute to AI in women, with the incidence after vaginal delivery reported as 3% or more (4). Nevertheless, studies investigating the impact factors contributing to AI other than delivery methods, such as epidural anesthesia, shoulder dystocia, and abnormal occiput position, are insufficient.

Therefore, this research aimed to explore antepartum and intrapartum risk factors for AI during the first year of postvaginal delivery by conducting a case control study. This project is proposed to provide a comprehensive understanding of the high-risk factors contributing to AI among women undergoing vaginal delivery.

## 2. Materials and methods

This case study was conducted on vaginally delivered women at Peking University Third Hospital between January 1, 2014, and June 30, 2018, consecutively. All participants were requested to complete a telephone interview 1 year after delivery. However, women without contact information, telephone number error, those who did not agree to answer questionnaires or answered the phone but did not complete a valid questionnaire with the interviewer were excluded.

During the telephone interview, every participant answered a questionnaire (Table 1) modified from Jorge and Wexner (7, 8) to review whether they had experienced AI within the first year after giving birth. The questionnaire assessed the AI type, frequency, and severity, including its impact on the quality of life. Afterward, obtained data were compiled into a scoring system to quantify the severity of AI. The symptoms included five categories: involuntary loss of flatus, liquid stool, solid stool, wearing a pad, and life style alteration. The frequency of symptoms was recorded as never, rarely (< l month), sometimes (<l week, ≥l month), usually (<l day, ≥l week), always (≥l day), and was scored separately as 0, 1, 2, 3, or 4. The scores for each category were added up to obtain a total score ranging from 0 (complete continence) to 20 (complete incontinence). Based on their total scores, participants were classified as having mild AI (score of 1–2), moderate AI (score of 3–9), or severe AI (score greater than 9) (9). Subsequently, all patients were requested to recall whether there were symptoms of AI at 6 weeks, 6 months, and 12 months after delivery, respectively, to calculate the prevalence of postpartum AI at different time points within 1 year after delivery.

**TABLE 1:** The Jorge and Wexner incontinence score.

Type of incontinence	Frequency				
	Never	Rarely	Sometimes	Usually	Always
Solid	0	1	2	3	4
Liquid	0	1	2	3	4
Gas	0	1	2	3	4
Wears pad	0	1	2	3	4
Lifestyle alteration	0	1	2	3	4

Never = 0; rarely is < 1/month; sometimes is < 1/week but ≥ 1/month; usually is < 1/day but ≥ 1/week; always is ≥ 1/day.

In all valid questionnaires, participants with scores above 0 were defined as having AI. Patients with AI during the first year after vaginal delivery were included in the study group. A control group was then selected at random from the group of participants who did not report AI symptoms. To ensure an adequate sample size and statistical power, the control group included twice as many participants as the study group. Pelvic floor rehabilitation is a non-invasive modality involving cognitive reeducation, bladder or bowel training, biofeedback, electrotherapy, and retraining of the pelvic floor and associated musculature (usually 5 weeks, twice a week) (10). After delivery, patients might receive pelvic floor rehabilitation in different settings, including hospitals, patients’ home, or for-profit medical institutions, where various approaches and techniques were employed. Therefore, to control for potential confounding biases, patients with postpartum rehabilitation and prenatal incontinence were excluded from the study. Medical data from the remaining participants in both the study and control groups were compared to identify the risk factors associated with postpartum AI.

The study collected medical data on pregnancy, labor, and neonates from the hospital database system. The variables of interest included maternal age, body mass index (BMI) before pregnancy, weight gain during pregnancy, parity, twin or triplet pregnancy, gestational diabetes mellitus (GDM) or diabetes mellitus (DM), hypertensive disorders complicating pregnancy (HP), gestational weeks, total infants’ birth weight, prolonged second-stage labor, epidural anesthesia, shoulder dystocia, abnormal occiput position, mode of delivery, mode of episiotomy, and different degrees of perineal tear. These factors were examined in the analysis to identify their association with the risk of postpartum AI.

The study also categorized maternal age into three groups: <30 years, ≥30 years but <35 years, and≥35 years old. According to the BMI classification in the Asian population (11), women with a BMI of 18.5–22.9 kg/m^2^ were considered normal, those with a BMI less than 18.5 kg/m^2^ were classified as thin, and those with a BMI of≥23 kg/m^2^ were overweight. Additionally, perineal tears were clinically classified into degrees 1–4 (12). Nevertheless, due to the small numbers of patients with degree III and degree IV perineal tears, they were combined into one category for analysis.

Statistical analyses were conducted using the SPSS 25.0 version. Continuous variables, such as weight gain during pregnancy and gestational weeks, were expressed as means and standard deviation (X+SD) and compared using a *t*-test. Categorical variables, such as age groups, GDM, etc., were expressed as numbers and proportions. Therefore, they were compared using the Chi-square or Fisher’s exact tests as appropriate. Furthermore, multivariable logistic regression was used to explore independent impact factors for postpartum AI. All variables that were found to be significantly associated with AI in the univariate analysis were included in the multivariable logistic regression model. A two-sided *p*-value of less than 0.05 was considered statistically significant.

After a multi-step screening process, the final risk factors were used to construct a nomogram for predicting the probability of AI postpartum. According to the regression coefficient, each variable that was included corresponded to a point at each value. A total point was equal to the sum of the points of all variables for each patient. The relationship between the total points and the probability of AI postpartum was visualized on the bottom of the nomogram.

Restricted cubic spline (RCS) was used to expore potential non-linear relationships between total infants’ birth weight as a continuous variable and AI postpartum. The RCS model used five knots (5th, 27.5th, 50th, 72.5th, and 95th percentile of total infants’ birth weight), and the relationship was plotted as odds ratio and 95% confidence interval (CI) for the risk of AI postpartum on the y-axis and birth weight on the x-axis.

The Research Ethics Committee of Peking University Third Hospital (IRB00006761-M2019350) approved and exempted the signing of the informed consent, and experiments were conducted following the Declaration of Helsinki as revised in 2013.

## 3. Results

### 3.1. Patient characteristics

During the 4 years and 6 months study period, 13,681 women at Peking University Third Hospital underwent vaginal delivery, out of which 10,957 (80.09%) responded to the telephone interview. Among these respondents, we detected 10,228 (74.76%) valid cases. While 223 (2.18%) women experienced AI symptoms during the first year after delivery, 210 (2.05%) had AI at 6 weeks postpartum, 171 (1.67%) at 6 months, and 102 (1.0%) at 12 months after delivery.

**FIGURE 1:**
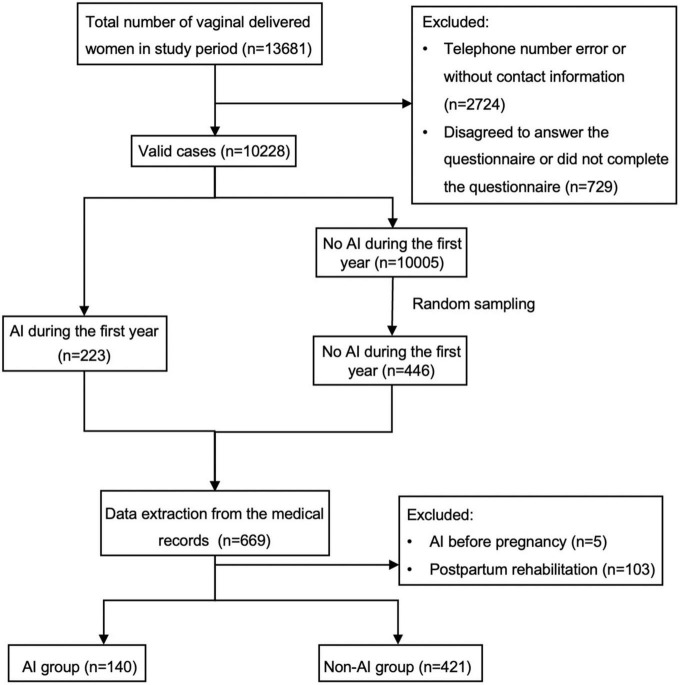
Flowchat of participants through the study. The two groups consisted of vaginal delivered women at Peking University Third Hospital between January 1, 2014 and June 30, 2018 consecutively, except those who didn’t get contact information or complete a valid questionnaire. The presence of anal incontinence (AI) was determined based on a Jorge and Wexner score greater than 0. The control group was randomly selected from women with a score of 0, and the number of control group participants was twice that of the study group. Women who had AI before pregnancy or pelvic floor rehabilitation postpartum were also excluded.

Subsequently, 446 women who did not experience AI were randomly selected for comparison with the 223 women. After excluding women who had AI before pregnancy or underwent pelvic floor rehabilitation postpartum, the AI group comprised 140 cases, while the none AI group included 421 cases during the first year after vaginal delivery (Figure 1).

All of 140 women were scored as the following: 80, 16, 7, 14, 9, 2, 11 women had a score of 1, 2, 3, 4, 5, 6, 7, respectively, and 1 case had a score of nine. Of the 140 women in AI group, 68.6% (96/140) experienced mild AI, and 31.4% (44/140) had moderate AI. None of them suffered from severe AI. Additionally, while 97.1% (136/140) women in the AI group had gas incontinence (involuntary loss of flatus), the percentages of liquid and solid incontinence (involuntary loss of liquid stool or solid stool) were 32.1% (45/140) and 15% (21/140), respectively. Furthermore, among the 140 cases with postpartum AI, 10 women gave birth to twins, 1 woman gave birth to triplets, and only 1 woman in the none AI group delivered twins.

### 3.2. Maternal risk factors associated with AI

Maternal risk factors associated with AI are presented in Table 2. We found that older maternal age at delivery, parity, and twins/triplets were significantly associated with AI postpartum (*p* < 0.05). Although the BMI before pregnancy showed a trend of increasing risk with AI, statistical significance was not observed (*p* = 0.06). Additionally, there were no statistical difference in GDM, DM, weight gain during pregnancy, and HP between the two groups.

**TABLE 2:** Maternal risk factors associated with AI^a^.

	AI (*n* = 140)	None AI (*n* = 421)	Total (*n* = 561)	t/Z/χ^2^	*P*
Maternal age (y)	–	–	–	9.27	0.01
<30	27 (19.3)	113 (26.8)	140 (25.0)	–	–
30–35	60 (42.9)	203 (48.2)	263 (46.9)	–	–
≥35	53 (37.9)	105 (24.9)	158 (28.2)	–	–
BMI before pregnancy (kg/m^2^)	–	–	–	5.61	0.06
<18.5	8 (5.7)	44 (10.5)	52 (9.3)	–	–
18.5–23	84 (60.0)	269 (63.9)	353 (62.9)	–	–
≥23	48 (34.3)	108 (25.7)	156 (27.8)	–	–
Weight gain during pregnancy (kg)	13.16 ± 2.72	13.30 ± 2.64	13.20 ± 2.70	0.54	0.59
Parity	–	–	–	7.87	<0.01
1	125 (89.3)	403 (95.7)	528 (94.1)	–	–
2	15 (10.7)	18 (4.3)	33 (5.9)	–	–
Twins or triplets	–	–	–	25.61	<0.01
No	129 (92.1)	420 (99.8)	549 (97.9)	–	–
Yes	11 (7.9)	1 (0.2)	12 (2.1)	–	–
GDM or DM	–	–	–	0.78	0.70
None	106 (75.7)	310 (73.6)	416 (74.2)	–	–
GDM	33 (23.6)	104 (24.7)	137 (24.4)	–	–
DM	1 (0.7)	7 (1.7)	8 (1.4)	–	–
HP	–	–	–	0.41	0.52
Yes	4 (2.9)	17 (4.0)	21 (3.7)	–	–
No	136 (97.1)	404 (96.0)	540 (96.3)	–	–

AI, anal incontinence; BMI, body mass index (calculated as weight in kilograms divided by height in meters squared); GDM, gestational diabetes mellitus; DM, diabetes mellitus; HP, hypertensive disorders complicating pregnancy.

^a^Values are given as mean ± standard deviation, or as number (percentages).

### 3.3. Risk factors during labor associated with AI

Risk factors during labor associated with AI are shown in Table 3. The risk of AI was significantly higher with increasing total infants’ birth weight, forceps-assisted vaginal delivery, midline episiotomy, and second to fourth-degree perineal tear (*p* < 0.01). However, there were no significant difference in the proportions of second stage prolonged (5.5 *vs.* 2.2%) and shoulder dystocia (1.4 *vs.* 0.2%) between the AI and none AI group (*p* > 0.05). Results also showed that gestational weeks at delivery, abnormal occiput position, and epidural anesthesia were not associated with AI (*p* > 0.05).

**TABLE 3:** Risk factors during labor associated with AI^a^.

	AI (*n* = 140)	None AI (*n* = 421)	Total (*n* = 561)	t/Z/χ^2^	*P*
Gestational weeks	39.55 ± 1.59	39.37 ± 1.53	39.41 ± 1.55	1.20	0.23
Total infants’ birth weight (g)	–	–	–	–	<0.01
Less than 2,500	0 (0.0)	15 (3.6)	15 (2.7)	–	–
2,500–2,999	5 (3.6)	78 (18.5)	83 (14.8)	–	–
3,000–3,499	29 (20.7)	197 (46.8)	226 (40.3)	–	–
3,500–3,999	41 (29.3)	111 (26.4)	152 (27.1)	–	–
4,000 or more	65 (46.4)	20 (4.8)	85 (15.2)	–	–
Second stage prolonged^b^	–	–	–	2.11	0.15
Yes	6 (5.5)	8 (2.2)	14 (2.9)	–	–
No	104 (94.5)	357 (97.8)	461 (97.1)	–	–
Epidural anesthesia	–	–	–	–	0.58^c^
Yes	0 (0.0)	3 (0.7)	3 (0.5)	–	–
No	140 (100.0)	418 (99.3)	558 (99.5)	–	–
Shoulder dystocia	–	–	–	–	0.16^c^
Yes	2 (1.4)	1 (0.2)	3 (0.5)	–	–
No	138 (98.6)	420 (99.8)	558 (99.5)	–	–
Abnormal occiput position	–	–	–	–	0.07^c^
Yes	7 (5.0)	8 (1.9)	15 (2.7)	–	–
No	133 (95.0)	413 (98.1)	546 (97.3)	–	–
Mode of delivery	–	–	–	35.74	<0.01
Spontaneous vaginal	116 (82.9)	409 (97.1)	525 (93.6)	–	–
Vaginal forceps	24 (17.1)	12 (2.9)	36 (6.4)	–	–
Episiotomy	–	–	–	–	<0.01^c^
No episiotomy	92 (65.7)	227 (53.9)	319 (56.9)	–	–
Midline episiotomy	7 (5.0)	3 (0.7)	10 (1.8)	–	–
Mediolateral episiotomy	41 (29.3)	191 (45.4)	232 (41.4)	–	–
Perineal tear	–	–	–	–	<0.01^c^
Intact perineum	44 (31.4)	195 (46.3)	239 (42.6)	–	–
First-degree perineal tear	76 (54.3)	218 (51.8)	294 (52.4)	–	–
Second-degree perineal tear	9 (6.4)	8 (1.9)	17 (3.0)	–	–
Third to fourth-degree perineal tear	11 (7.9)	0 (0)	11 (2.0)	–	–

AI, anal incontinence.

^a^Values are given as mean ± standard deviation, or as number (percentages).

^b^Includes only those women who experienced second-stage labor (n = 475).

^c^Fisher’s exact test.

### 3.4. Multivariate logistic analysis of risk factors associated with AI

**FIGURE 2:**
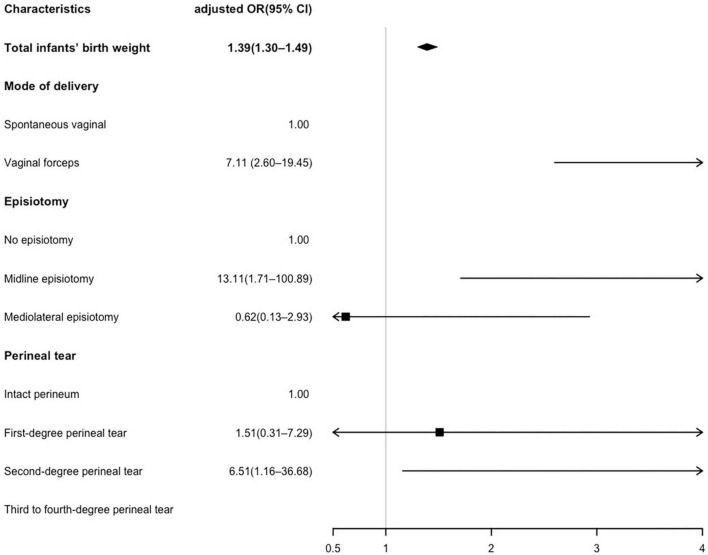
Multivariate logistic analysis of risk factors associated with anal incontinence (AI). Maternal age, body mass index (BMI), abnormal occiput position and parity were adjusted in the logistic regression model. The unit of total infants’ birth weight is 100 g.

The multivariable logistic regression of risk factors for AI is shown in Figure 2. Maternal age, BMI before pregnancy, abnormal occiput position, parity, total infants’ birth weight, mode of delivery, episiotomy, and perineal tear were included in the logistic regression model. However, since a significant correlation was observed between twins or triplets and infants’ birth weight (*p* < 0.01), twins or triplets were excluded from the logistic regression model. The results showed that every 100 g gain in infants’ birth weight significantly increased the risk of AI (*OR* 1.39, *95% CI* 1.30–1.49), and forceps-assisted vaginal delivery was associated with a 7-fold risk of AI (*OR* 7.11, *95% CI* 2.60–19.45). Compared with the no episiotomy subgroup, midline episiotomy increased the risk of AI (*OR* 13.11, *95% CI* 1.71–100.89). However, mediolateral episiotomy did not show a protective effect (*OR* 0.62, *95% CI* 0.13–2.93) for preventing postpartum AI. Additionally, a second-degree perineal tear was associated with a higher risk of AI (*OR* 6.51, *95% CI* 1.16–36.68) compared to an intact perineum.

### 3.5. Clinical utility of a nomogram

**FIGURE 3:**
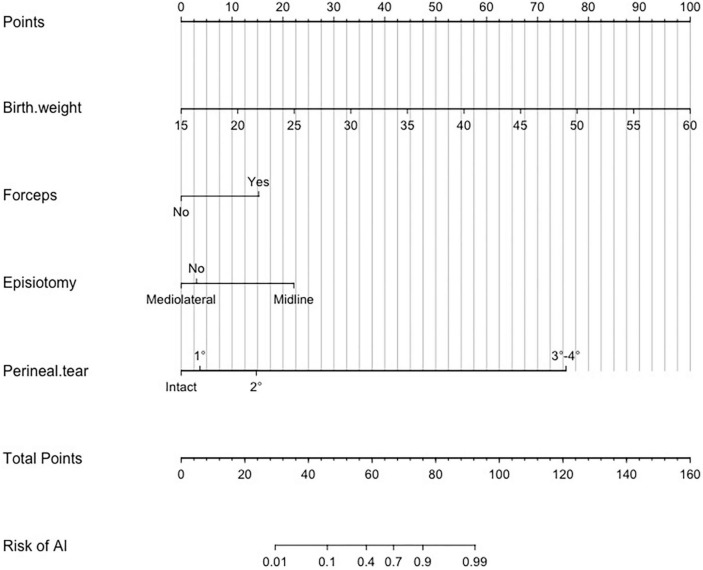
Nomogram to estimate the risk of anal incontinence (AI) 1 year after vaginal delivery. To use the nomogram, find the position of each variable on the corresponding axis, draw a line to the points axis for the number of points, add the points from all of the variables, and draw a line from the total points axis to determine the AI probabilities at the lower line of the nomogram. The unit of birth weight is 100 g. For example, if a woman had a fetal birth weight of 2,700 g, underwent forceps delivery, received mediolateral episiotomy, and had second-degree perineal tear, the point values for each of these risk factors would be 28, 15, 0, and 15, respectively. The total points were 58, which corresponded to a 40% risk of postpartum AI.

Based on the results of the multivariate logistics regression analysis, we further constructed a nomogram by combining prognostic factors including total infants’ birth weight, forceps assisted, episiotomy, and perineal tear (Figure 3). A quantitative method was made accessible for clinicians to predict the probability of AI during the first year after vagianl delivery. Each risk factor was assigned a point value based on its contribution to the overall risk of AI, and the total score corresponded to the predicted probability of AI. For example, a pregnant woman with fetal birth weight of 2,700 g, utilizing forceps, employing mediolateral episiotomy and with second-degree perineal tear would receive 28, 15, 0, and 15 points, respectively, resulting in a total score of 58 and a predicted probability of 40% for AI. Clinicians can use this tool to assess a woman’s individual risk of AI and provide appropriate management and preventative strategies.

### 3.6. Restrictive cubic spline model

**FIGURE 4:**
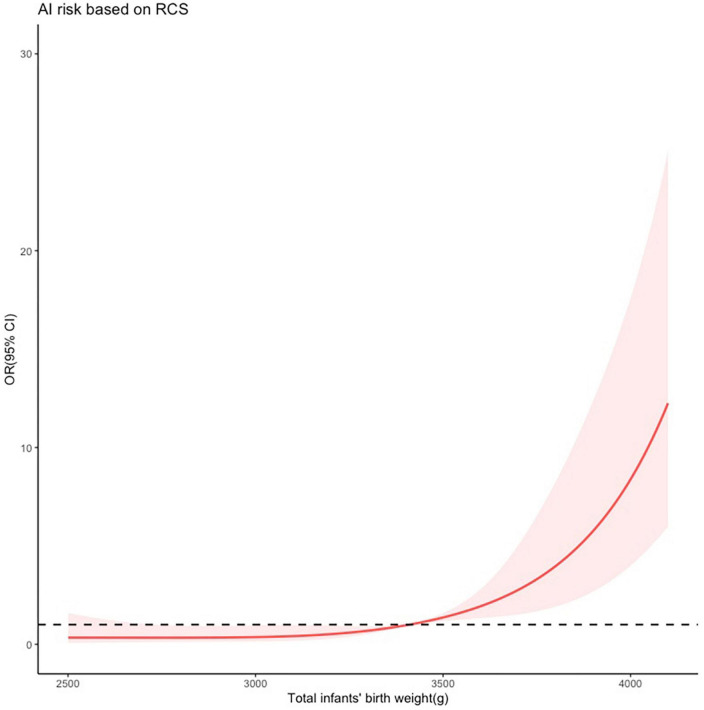
Restricted cubic spline modeling of the relationship between total infants’ birth weight and the risk of anal incontinence (AI) 1 year after vaginal delivery. The odds ratios derived from a multivariate regression model is shown on the y-axis. The 95% confidence intervals (Cis) of the adjusted odds ratios are represented by the shaded area. The dotted line represents that the odds ratio is 1. The risk function demonstrates an inflection point at 3,400 g.

The restricted cubic spline analysis (Figure 4) was applied to further investigate the relationship between birth weight and the risk of AI postpartum. Following adjustment for forceps-assisted delivery, episiotomy and perineal tear, the analysis revealed a positive association between birth weight and the risk of AI postpartum. Specifically, when the birth weight exceeded 3,400 g, the risk of AI increased dramatically. Moreover, when the birth weight exceeded 4,000 g, the risk increased by a factor of approximately 10.

## 4. Discussion

In this study, we observed a decrease in the prevalence of AI within 1 year after vaginal delivery. The incidence rates were 2.05, 1.67, and 1.0% at 6 weeks, 6 months, and 1 year after vaginal delivery, respectively. Visible natural descent trends were consistent with Meyer’s results (13). Additionally, the incidence of postpartum AI changed with the timing after delivery, which supported the idea of self-recovery of pelvic floor function. Due to individual privacy concerns, some patients were hesitant to share their anus-related issues, which may have resulted in a lower incidence rate of postpartum AI in our study than the actual rate. Hence, we considered that compared with face-to-face or telephone interviews, postal-interview surveys could lead to a higher AI incidence rate (4). Our results also indicated that postpartum AI was predominantly mild, accounting for two-thirds of all cases. However, in terms of the types of incontinence, the proportion of pure gas incontinence was the highest, while the proportion of liquid and solid incontinence was much lower, consistent with Eason’s study fingdings (14).

Subsequently, the RCS was used to evaluate total birth weight for predicting postpartum AI. Results showed that the risk of AI increased dramatically when the birth weight exceeded 3,400 g. This result was consistent with previous studies (15), which identified a birth weight of 4,000 g or more as a risk factor for AI after vaginal delivery. It is worth noting that human weight is related to race, which results in varying criteria for defining overweight and obesity across different countries. For instance, the mean BMI in China was 23.9 kg/m^2^ in 2014, while in the US, UK, and Australia, it was 28.8, 27.3, and 27.2 kg/m^2^, respectively (16). In this study, we observed that total infants’ birth weight of more than 3,400 g increased the risk of postpartum AI, which can be attributed to the lower mean BMI of the Chinese population. We also observed that twin or triplet pregnancies significantly increased the risk of AI, which could be explained by the greater total infants’ birth weight. High birth weight can contribute to AI by compressing, stretching, or tearing nerves, muscles, and connective tissues. Thus we used the sum of fetal weight rather than the average fetal weight in cases of twins or triplets. Therefore, nutrition management and appropriate control of infant weight growth during pregnancy are essential to prevent postpartum AI.

In addition, this study identified forceps delivery (17.1% for AI *vs.* 2.9% for none AI) as an independent risk factor for postpartum AI among the intrapartum factors. This result was consistent with most prior studies, which demonstrated that forceps delivery substantially increased the risk of AI compared to vaginal delivery (17–19). However, there was no significant relationship between vacuum extraction and postpartum AI (20). Forceps delivery not only causes muscle lacerations, such as the anal sphincter, levator anus, and injury of surrounding fascia tissue easily, it also impairs muscle contraction function and maintenance of fascia tension. Therefore, clinicians should exercise caution when considering the use of forceps during delivery, particularly for pregnant women with high-risk factors, such as macrosomia. However, if forceps are necessary to assist delivery, careful monitoring for AI symptoms after delivery and timely pelvic floor rehabilitation training to prevent AI are advised.

Another important intrapartum factor was perineal laceration that increased the risk of postpartum AI. Our study found that after adjusting for variables such as parity and BMI, second to fourth-degree perineal tears also increased the risk of AI within 1 year postpartum compared to intact perineum. This finding was consistent with previous studies, which have identified anal sphincter injury as the most common cause of postpartum AI (18, 21–24). To reduce the risk of AI, protecting the perineum during delivery is crucial. In cases where perineal laceration occurs, it is essential to suture the wound promptly to prevent infection and avoid the development into old laceration, thus minimizing the occurrence of postpartum AI.

It is important to note that the effects of episiotomy on postpartum AI may differ depending on the type of episiotomy performed. Most studies have observed midline episiotomy as an independent risk factor for OASIS (25, 26). In this study, midline episiotomy was found to be a significant risk factor for postpartum AI, while mediolateral episiotomy did not show a statistically significant correlation. Nevertheless, among the women who suffered postpartum AI, 29.3% of them received mediolateral episiotomy, while among the women who did not suffer postpartum AI, 45.4% of them received mediolateral episiotomy, suggesting that mediolateral episiotomy had a protective tendency for postpartum AI. Currently, there is still a debate about the effect of midline and mediolateral episiotomy on postpartum AI (20). Some studies have reported that increasing the angle of lateral cutting from the midline distance by 6^°^ can reduce the risk of III-degree perineal tear in postpartum women by around 50% (25, 26). However, many studies did not differentiate between midline and mediolateral episiotomy, making it difficult to analyze protective effects or risks separately. Therefore, it is crucial to distinguish between different types of perineotomy and analyze them separately to provide better guidance for clinicians.

This study encountered some limitations, mainly including the following aspects: (1). The study was a 1 year postpartum telephone follow-up for all parturients. Even though many AI studies have used this method and the credibility has been verified, the positive rate of telephone follow-up AI was slightly lower than the email follow-up. (2). Due to the limitation of sample size, this study did not make a hierarchical analysis of multiparae and primiparae, but used a multifactor model to adjust its impact. (3). Due to the limitation of research time, the length of this study was only 1 year after delivery.

In conclusion, postpartum AI can have a severe impact on the lives and psychological wellbeing of women following vaginal delivery. Our study found that certain factors, such as infants’ birth weight of 3,400 g or more, forceps-assisted vaginal delivery, midline episiotomy, and second to fourth-degree perineal tear, increased the risk of AI within the first year after delivery. Thus, women are advised to take nutrition management and aim for a proper birth weight. In the process of delivery, both forceps and midline episiotomy are serious but avoidable risk factors for AI, especially midline episiotomy. The decision to perform episiotomy is heavily dependent on the opinion of the obstetricians and is based on the clinical scenario at the time of delivery. In most cases, episiotomy is not necessary. Obstetricians should limit the routine use of episiotomy and prioritize protecting the perineum from severe laceration. When deemed necessary, mediolateral episiotomy is preferred due to its potential profective effect on postpartum AI.

## Data availability statement

The original contributions presented in this study are included in the article/supplementary material, further inquiries can be directed to the corresponding authors.

## Ethics statement

The studies involving human participants were reviewed and approved by the Research Ethics Committee of Peking University Third Hospital (IRB00006761-M2019350). The patients/participants provided their written informed consent to participate in this study.

## Author contributions

YJ: data collection and analysis and manuscript writing. QL: data analysis and manuscript writing. LZ: study designing, statistical analysis, and manuscript writing. YW: project development. All authors contributed to the article and approved the submitted version.
